# Braithwaite’s Retrospect

**Published:** 1874-08

**Authors:** 


*Braithwaite's Restrospect of Practical Medicine and Surgery.* Part LXIX. New York: W. A. Townsend, 1874.

Braithwaite's Retrospect still continues semi-annually to make its appearance upon the office table of a large number of American physicians. This admirable Journal has been so long and favorably known by the profession that it would seem like loosing an old friend to have it omit its visits. The idea has become quite prevalent that the publication of this Journal was about to be suspended; this arose, doubtless, from the announcement of the suspension of Rankin's Half Yearly Abstract.

